# Staging the axilla in breast cancer patients with ^18^F-FDG PET: how small are the metastases that we can detect with new generation clinical PET systems?

**DOI:** 10.1007/s00259-014-2689-7

**Published:** 2014-02-22

**Authors:** Dimitri Bellevre, Cécile Blanc Fournier, Odile Switsers, Audrey Emmanuelle Dugué, Christelle Levy, Djelila Allouache, Cédric Desmonts, Hubert Crouet, Jean-Marc Guilloit, Jean-Michel Grellard, Nicolas Aide

**Affiliations:** 1 Nuclear Medicine Department, François Baclesse Cancer Centre, Avenue Général Harris, 14076 Cedex 5, Caen France; 2 Pathology Department, François Baclesse Cancer Centre, Caen, France; 3 Breast Cancer Unit, François Baclesse Cancer Centre, Caen, France; 4 Biostatistics and Clinical Research unit, François Baclesse Cancer Centre, Caen, France; 5 Radiation Oncology Department, François Baclesse Cancer Centre, Caen, France; 6 Medical Physics Department, University Hospital, Caen, France; 7 Surgical Oncology, François Baclesse Cancer Centre, Caen, France; 8 Normandie Université, Caen, France

**Keywords:** PET/CT, Breast cancer, Axillary staging, Fluorodeoxyglucose, PSF reconstruction

## Abstract

**Purpose:**

Point spread function (PSF) reconstruction improves spatial resolution throughout the entire field of view of a PET system and can detect smaller metastatic deposits than conventional algorithms such as OSEM. We assessed the impact of PSF reconstruction on quantitative values and diagnostic accuracy for axillary staging of breast cancer patients, compared with an OSEM reconstruction, with emphasis on the size of nodal metastases.

**Methods:**

This was a prospective study in a single referral centre in which 50 patients underwent an ^18^F-FDG PET examination before axillary lymph node dissection. PET data were reconstructed with an OSEM algorithm and PSF reconstruction, analysed blindly and validated by a pathologist who measured the largest nodal metastasis per axilla. This size was used to evaluate PET diagnostic performance.

**Results:**

On pathology, 34 patients (68 %) had nodal involvement. Overall, the median size of the largest nodal metastasis per axilla was 7 mm (range 0.5 – 40 mm). PSF reconstruction detected more involved nodes than OSEM reconstruction (*p* = 0.003). The mean PSF to OSEM SUV_max_ ratio was 1.66 (95 % CI 1.01 – 2.32). The sensitivities of PSF and OSEM reconstructions were, respectively, 96 % and 92 % in patients with a largest nodal metastasis of >7 mm, 60 % and 40 % in patients with a largest nodal metastasis of ≤7 mm, and 92 % and 69 % in patients with a primary tumour ≤30 mm. Biggerstaff graphical comparison showed that globally PSF reconstruction was superior to OSEM reconstruction. The median sizes of the largest nodal metastasis in patients with nodal involvement not detected by either PSF or OSEM reconstruction, detected by PSF but not by OSEM reconstruction and detected by both reconstructions were 3, 6 and 16 mm (*p* = 0.0064) respectively. In patients with nodal involvement detected by PSF reconstruction but not by OSEM reconstruction, the smallest detectable metastasis was 1.8 mm.

**Conclusion:**

As a result of better activity recovery, PET with PSF reconstruction performed better than PET with OSEM reconstruction in detecting nodal metastases ≤7 mm. However, its sensitivity is still insufficient for it to replace surgical approaches for axillary staging. PET with PSF reconstruction could be used to perform sentinel node biopsy more safely in patients with a primary tumour ≤30 mm and with unremarkable PET results in the axilla.

**Electronic supplementary material:**

The online version of this article (doi:10.1007/s00259-014-2689-7) contains supplementary material, which is available to authorized users.

## Introduction

Breast cancer is the most frequent malignancy in women in Western countries and the second leading cause of cancer-related deaths among women. Axillary lymph node status is the most important prognostic factor for recurrence and survival. Therefore, accurate staging at the time of initial diagnosis is crucial. PET using ^18^F-FDG has been used for staging, restaging and therapy monitoring in a variety of cancer types, including breast cancer [1–5]. In newly diagnosed breast cancer, ^18^F-FDG PET is not recommended for routine staging of axillary lymph nodes because its sensitivity is too low [1]. A recent systematic review found that the sensitivity of PET/CT systems in the detection of axillary nodal metastases ranges from 44 % to 67 % [6]. This low sensitivity is due in part to the limited spatial resolution of PET systems, leading to partial volume effects (PVE) that cause significant underestimation of the radioactivity concentration in lesions smaller than two to three times the spatial resolution of the system. Consequently, small cancer deposits and especially micrometastases (<2 mm) are very unlikely to be detected.

In recent years, major hardware and software improvements have been implemented in PET imaging. In particular, advanced reconstruction algorithms that model the point spread function (PSF) of a system have recently become commercially available [7, 8]. PSF reconstruction improves spatial resolution throughout the entire field of view (FOV), reduces PVE and improves image contrast. As a result, newer-generation clinical PET systems equipped with such algorithms can be expected to detect small-volume metastases better.

The aim of this prospective study in single referral centre was to evaluate the impact of PSF reconstruction on quantitative values and the diagnostic accuracy of ^18^F-FDG PET for the axillary staging of breast cancer patients, as compared with a conventional algorithm, OSEM (ordered subsets expectation maximization). PET results were compared with pathological results with special emphasis on the size of nodal metastases.

## Materials and methods

### Study design

This single-centre prospective study was approved by the local Ethics Committee (CPP Nord-Ouest III, reference 2009-10). Informed and signed consent was obtained from all patients. Patients with newly diagnosed, histologically proven breast cancer for which breast surgery plus axillary lymph node dissection (ALND) was indicated were included from April 2009 to June 2012. ALND was indicated for a tumour of >30 mm and/or for multifocal disease, or for suspected nodal involvement on physical examination. None of the patients had received neoadjuvant chemotherapy. ^18^F-FDG PET/CT was used for pretherapeutic staging of patients. The OSEM algorithm and PSF reconstruction were used to reconstruct all PET data, but only PSF reconstruction was used in the patients’ diagnostic work-up.

### PET/CT scanning

PET imaging studies were performed on a Biograph TrueV (Siemens Medical Solutions) containing a six-slice spiral CT component. The TrueV system has been described in detail elsewhere [9]. ^18^F-FDG injection was preceded by a 6-h fasting period and a 15-min rest in a warm room. Mean ± SD injected activity was 4.1 ± 0.5 MBq per kg of body weight. Patients were scanned 60 ± 5 min after ^18^F-FDG injection from the skull base to the mid-thighs using the following parameters:

- CT acquisition: 60 mAs, 130 kVp, pitch 1, and 6 × 2 mm collimation
- PET emission: 3-D mode, 2 min 40 s per bed position in patients of low and average weight, or 3 min 40 s per bed position in overweight patients
- PET reconstruction: OSEM 3-D algorithm (four iterations and eight subsets) and PSF algorithm (HD; TrueX, Siemens Medical Solutions; three iterations and 21 subsets)

A 5-mm gaussian filter was applied to the OSEM images, but no postfiltering was used in the PSF images because PSF reconstruction has been shown to achieve maximal performance with little or no filtering [10]. These reconstruction parameters were as recommended by Siemens Healthcare for whole-body PET/CT scan oncological reading, and the OSEM parameters meet the EANM requirements regarding activity recoveries when scanning the National Electrical Manufacturers Association (NEMA) NU 2 phantom as per EANM standards of procedure [11]. For both reconstructions, the matrix size was 168^2^, resulting in a 4.07×4.07×5 mm voxel size. Scatter and attenuation corrections were applied.

In order to evaluate the spatial resolution of our PET system using a geometry similar to a clinical PET examination, i.e. at a location in the FOV similar to that of the axilla, linear sources were imaged on both sides of the NEMA NU 2 phantom at a 15-cm radial offset. These sources were capillaries (inner diameter 1 mm) filled with a 70 or 87 MBq/cm^3^ ^18^F-FDG solution. The phantom was filled with a 20 kBq/cm^3^ ^18^F-FDG solution, as recommended by the EANM guidelines for PET tumour imaging [11]. Acquisition and reconstruction parameters were similar to those of a clinical PET scan described above. Full-width at half-maximum (FWHM) of the PSF was determined in the radial direction along profiles passing through the distribution peak, as recommended by the NEMA NU 2-2001 standard [12].

### PET/CT interpretation

All nuclear medicine physicians involved in this study had more than 2 years experience reading PET/CT images reconstructed with a PSF algorithm. After randomization, anonymize PET/CT examinations were reviewed on an eSoft/TrueD workstation (Siemens Medical Solutions) by a board-certified nuclear medicine physician (N.A.) who was not informed of patient outcome. With each reconstruction algorithm, the reader qualitatively interpreted axillary lymph node involvement defined as uptake superior to background noise, and the number of positive nodes in each PET examination. A second reader (D.B.) analysed all PET datasets to extract PET quantitative values for OSEM and PSF reconstructions as follows. Circular two-dimensional (2-D) regions of interest (ROIs) were drawn over axillary nodes considered to have pathologically increased uptake. ROIs were drawn on the axial slice where nodes displayed the highest ^18^F-FDG uptake. A 1-cm circular 2-D ROI was drawn over the pectoralis major at the level of the acromioclavicular joint to assess regional background uptake. ROIs were placed at exactly the same position on both OSEM and PSF images by using an ROI copy/paste function. The maximum and mean pixel values were extracted from each ROI. Maximum SUVs (SUV_max_) as well as node/background (N/B) ratios were computed as follows:

$$ SUV=\frac{ tumour\kern0.5em  activity\;\left( Bq/ cc\right)\times body\kern0.5em  weight\;(g)}{ injected\kern0.5em  dose\;(Bq)}, $$

$$ \raisebox{1ex}{$N$}\!\left/ \!\raisebox{-1ex}{$B$}\right.\kern0.5em  ratio=\frac{ Maximal\kern0.74em  node\kern0.74em  activity\kern0.34em \left( Bq/ cc\right)}{ Mean\kern0.62em  pectoralis\kern0.74em  activity\kern0.36em \left( Bq/ cc\right)}. $$

Finally, the length of the short axis (millimetres) as determined on CT slices was recorded for each axillary lymph node.

### Histological diagnosis

All but 3 patients underwent breast surgery with ALND within a month of PET imaging. The median delay between PET imaging and surgery was 7 days (range 1 – 56 days). After formalin fixation and paraffin embedding of the entire node, histological slides (4-μm thick) were stained with haematoxylin and eosin for histopathological examination. All pathological specimens were analysed by the same pathologist who measured the greatest diameter of each nodal metastatic deposit.

### Data analysis and statistical analysis

#### Quantitative data analysis

Quantitative data are presented as mean ± standard deviation (SD) or median (min−max) if necessary. The numbers of involved nodes detected by the PSF and OSEM algorithms were compared using the Wilcoxon rank test for paired samples. The sizes of the largest nodal metastasis per axilla depending on the PET status for both algorithms (PSF+/OSEM+: nodal involvement detected by both algorithms; PSF+/OSEM−: nodal involvement detected by PSF but not by OSEM; and PSF−/OSEM− nodal involvement not detected by either algorihtm) were compared using the Kruskal-Wallis rank sum test. The situation PSF−/OSEM+ (nodal involvement detected by OSEM but not by PSF) did not occur. A two-tailed *p* value less than 0.05 was considered statistically significant. The relationship between PSF and OSEM quantitative values was assessed using Bland-Altman plots.

#### Diagnostic performance evaluation

A positive PET/CT examination confirmed by pathology was considered as true-positive and otherwise as false-positive. A negative PET examination with no histological nodal involvement was considered as true-negative and if there was metastatic invasion on pathology as false-negative. Sensitivity, specificity, positive and negative predictive values, and accuracy were obtained on a per-patient basis for OSEM and PSF PET/CT with the 95 % confidence intervals. Positive and negative likelihood ratios (LR+, LR−) were computed for both algorithms:LR+ = sensitivity/(1 − specificity), LR− = (1 − sensitivity)/specificity. The LR incorporates the sensitivity and specificity of a test into a single measure. The LR− of a test is the probability of a patient who has the disease testing negative divided by the probability of a patient who does not have the disease testing negative. The best test to rule out a disease is the one with the smaller LR−. Likewise, the best diagnostic test to detect the disease is the one with the larger LR+ [13, 14].

Among metastatic patients, two groups were defined according to the size of the largest metastasis measured on pathology. The cut-off (7 mm) was the median size of all nodal metastatic deposits. We also evaluated the diagnostic performance of both algorithms in patients in whom the size of the primary tumour was ≤30 mm versus those patients in whom the size of primary tumour was >30 mm, a size above which the risk of macrometastases in the axilla is higher [15]. For this analysis, only the largest tumour was considered in patients with multifocal disease. The diagnostic performance achieved by the two algorithms were compared in each subgroup using the graphic representation proposed by Biggerstaff [16]. Briefly, to compare two diagnostic procedures, sensitivities, specificities or LRs are evaluated separately or using a summary statistic (e.g. Youden’s index). The graphical comparison proposed by Biggerstaff was chosen because it summarizes all these measures in one plot and is associated with a simple decision rule deciding if one test is superior for confirming the absence and/or presence of disease. In practice, the point that determines the true-positive and false-positive rates of a diagnostic procedure is plotted, e.g. on a ROC curve. Two lines are then created by connecting this point to points (0,0) and (1,1) separately. Thus, the LR+ is represented by the slope of the first line, and the LR− by the slope of the second line. Finally, the true-positive and false-positive rates of a second diagnostic procedure are plotted to determine which area among the four created by these lines it belong to.

Prism (GraphPad software) and Vassar University clinical research calculators (http://www.vassarstats.net) were used for graphs and statistics.

## Results

### Phantom acquisitions

For evaluation of the spatial resolution of the PET system with PSF and OSEM reconstructions, rather than computing FWHM in air as recommended in the NEMA standards, we evaluated FWHM in the geometry of a human breast and axilla PET examination by placing linear sources at a 15-cm radial offset on both sides of an anthropomorphic phantom filled with an activity in the range of the average activity expected in a human body.

Radial FWHM was measured on four linear sources and ranged from 2.35 mm to 2.48 mm for PSF reconstruction and from 6.19 mm to 6.46 mm for OSEM reconstruction.

### Patient demographics

Amongst the 55 patients included in this study accrued from April 2009 to June 2012, five were excluded from the analysis. The causes of exclusion were as follows: hyperglycaemia >3 g/L at the time of PET examination in one patient, decision to treat with neoadjuvant chemotherapy in one, metastatic disease requiring chemotherapy in two, and PET not possible prior to surgery in one. The tumour subtypes confirmed on histopathology included 43 infiltrating ductal carcinomas, two infiltrating lobular carcinomas, four mixed ductal/lobular infiltrating carcinomas and one infiltrating undifferentiated carcinoma. Patient characteristics are presented in Table 1; refer to the Supplementary material for more detailed data).

**Table 1:** Patient characteristics

Characteristic	Number (%) of patients
Clinical tumour stage	
Tx	1 (2)
T1	10 (20)
T2	27 (54)
T3	9 (18)
T4	3 (6)
Clinical node stage	
N0	28 (56)
N1	22 (44)
Oestrogen receptor status	
Positive	43 (86)
Negative	7 (14)
Progesterone receptor status	
Positive	33 (66)
Negative	17 (34)
HER-2/neu status	
Positive	7 (14)
Negative	43 (86)
Triple-negative	6 (12)
Histological grade status	
I	6 (12)
II	23 (46)
III	21 (42)
Size of tumour (cm)	
≤3	29 (58)
>3	21 (42)

### Pathological results

Pathology confirmed axillary lymph node involvement in 34 of 50 axillae. Of 782 resected lymph nodes, 151 showed involvement and 35 of these showed capsular invasion. Patients with lymph node involvement had an average of 4.4 metastatic lymph nodes on ALND. Overall, the median size of nodal metastases was 7 mm (0.5 – 40 mm).

### Impact of PSF reconstruction on quantitative values

#### SUV in nodal metastases

**Fig. 1:**
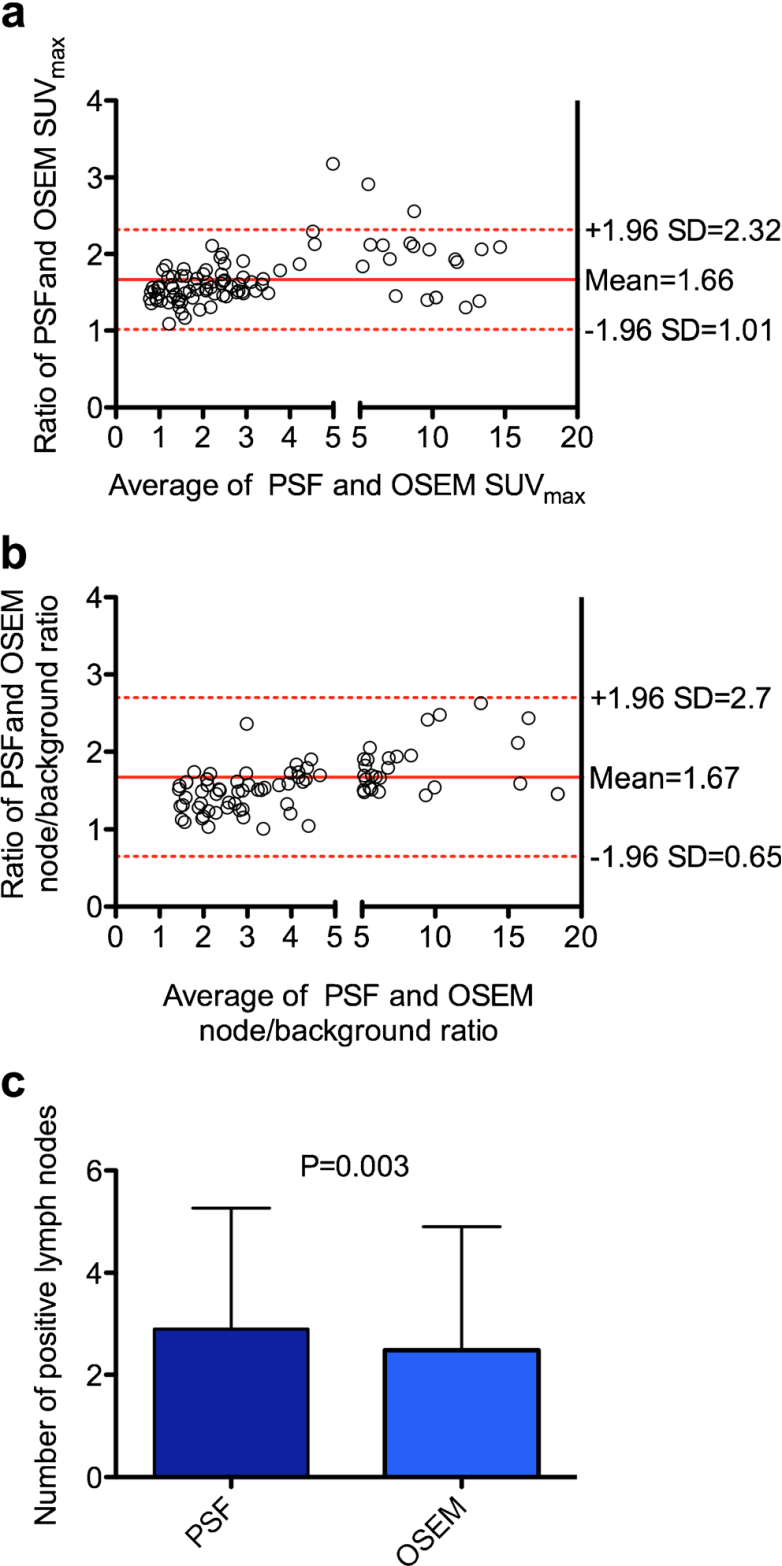
Impact of PSF reconstruction on quantitative values and number of detected nodes. Bland and Altman analysis for SUV_max_ (**a**) and node/background ratio (**b**) and the numbers of involved nodes accurately detected by PET with PSF reconstruction and OSEM reconstruction (**c**) are shown

We measured SUV on the PSF and OSEM reconstructions by drawing ROIs over 93 axillary or retropectoral lymph nodes in the 34 metastatic patients in whom PET showed nodes considered to have pathologically increased uptake of ^18^F-FDG. Of these 93 lymph nodes, 72 (77.4 %) had a short axis <1 cm, and their mean short-axis diameter was 0.82 ± 0.43 cm and their overall, mean SUV_max_ values for the PSF and OSEM reconstructions were 4.50 ± 4.39 and 2.60 ± 2.44 cm, respectively. The mean ratio between SUV_max_ measured on the PSF reconstructions and that on the OSEM reconstructions was 1.66 with narrow 95 % limits of confidence as shown on the Bland-Altman plot (Fig. 1a).

#### Node/background ratio

The mean ratio between node/background ratios measured on PSF reconstructions and those obtained on OSEM reconstructions was 1.67 with wider 95 % limits of confidence as compared with the SUV_max_ analysis (Fig. 1b).

### Impact of PSF reconstruction on diagnostic performance

**Fig. 2:**
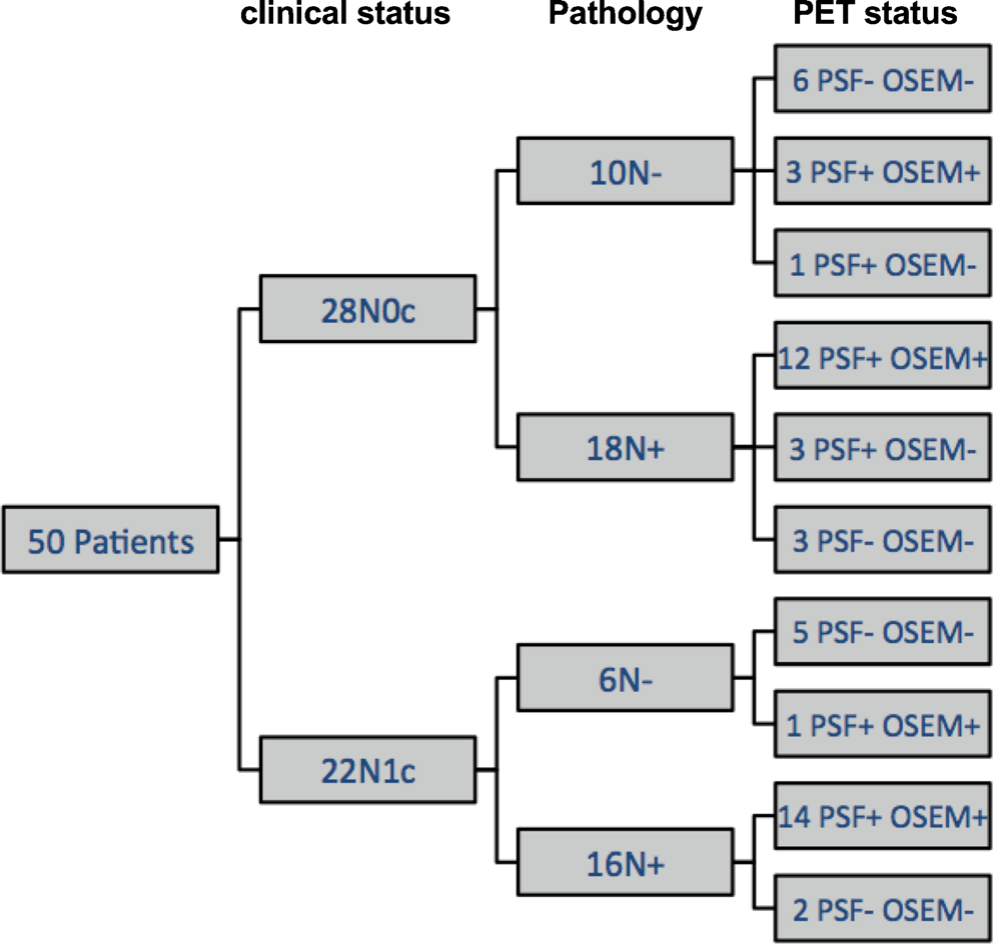
Flow-chart of clinical, pathological and PET status in 50 patients analysed (*PSF-*/*PSF+* negative/positive by PSF reconstruction, *OSEM-*/*OSEM+*) negative/positive by OSEM reconstruction

**Fig. 3:**
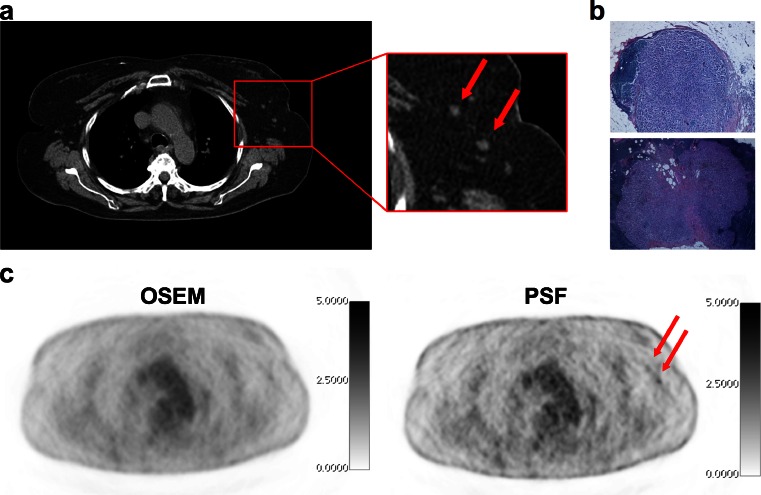
A 64-year-old patient with grade II infiltrating ductal carcinoma initially classified as T2N0c, in whom PSF reconstruction was positive and OSEM reconstruction was negative. Pathology revealed 4-mm and 6-mm lymph node metastases*red arrows*. Both OSEM and PSF reconstructions are scaled to the same maximum value (**a** CT images, **b** pathological specimen (HES staining), **c** corresponding PET slices)

**Fig. 4:**
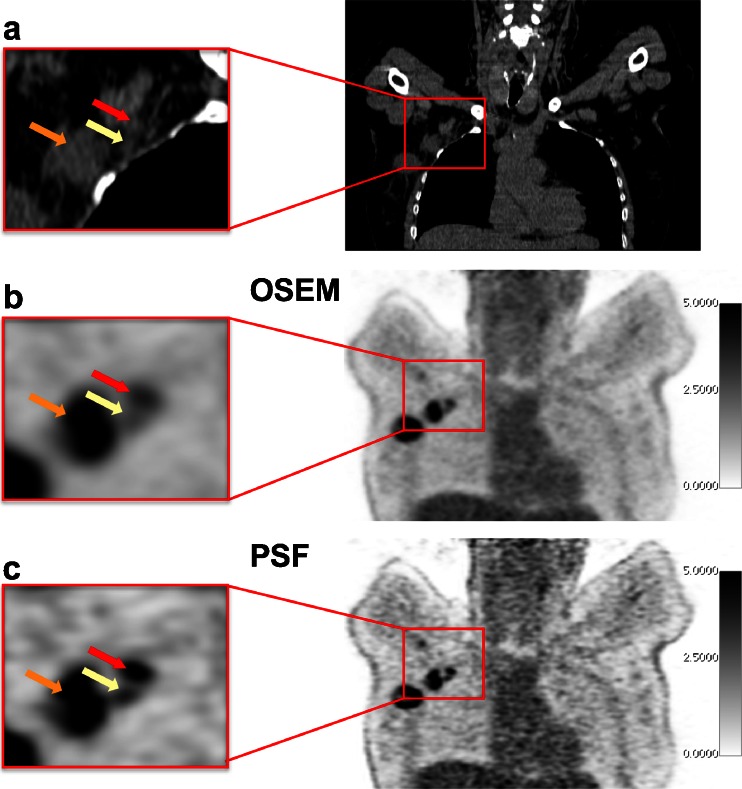
A 51-year-old patient with grade III infiltrating ductal carcinoma initially classified as T3N0c, in whom both PSF reconstruction and OSEM reconstruction were positive, but PSF reconstruction depicted an additional involved lymph node (*yellow arrows*) (**a** CT images, **b** PSF reconstruction, **c** OSEM reconstruction). Both OSEM and PSF reconstructions are scaled to the same maximum value. Note how PSF reconstruction improves activity recovery in small lesions (*red and yellow arrows*) as compared with the largest node (*orange arrows*)

PSF and OSEM reconstruction gave false-negative results in five and eight patients, respectively. Amongst the five patients with false-negative results by both PSF and OSEM reconstruction, four had just one lymph node involved with a median metastatic deposit size of 3 mm (3 – 7 mm). The results with PSF and OSEM reconstruction were concordant in 46 patients and discordant in 4 patients in whom PSF reconstruction was positive (three true-positive and one false-positive) and OSEM reconstruction was negative. It is noteworthy that the three patients in whom PSF reconstruction was true-positive and OSEM false-negative occurred showed clinical stage N0 (Fig. 2). Figure 3 illustrates a patient in which PSF reconstruction was true-positive, whereas OSEM reconstruction was false-negative. Figure 4 shows a patient in whom both reconstructions were true-positive, but more nodes were detected by PSF reconstruction.

PSF reconstruction detected more involved nodes (93) than OSEM reconstruction (83; *p* = 0.003; Fig. 1c). OSEM reconstruction detected axillary lymph node metastases with 76 % sensitivity and 75 % specificity, whereas PSF reconstruction achieved a better sensitivity (85 %) but a slightly lower specificity (69 %; Table 2).

**Table 2:** Diagnostic performance of PSF and OSEM reconstructions for axillary staging

	All patients		Breast tumour ≤30 mm		Breast tumour >30 mm	
	OSEM	PSF	OSEM	PSF	OSEM	PSF
Sensitivity (%)	76 (58 – 89)	85 (68 – 94)	69 (39 – 90)	93 (62 – 100)	81 (57 – 94)	81 (57 – 94)
Specificity (%)	75 (47 – 92)	69 (41 – 88)	70 (35 – 92)	60 (27 – 86)	83 (36 – 99)	83 (36 – 99)
Positive predictive value (%)	87 (68 – 96)	85 (68 – 94)	75 (43 – 93)	75 (47 – 92)	94 (71 – 100)	94 (71 – 100)
Negative predictive value (%)	60 (36 – 80)	69 (41 – 88)	64 (32 – 88)	86 (42 – 99)	56 (23 – 85)	56 (23 – 85)
Accuracy (%)	76 (62 – 86)	80 (66 – 89)	70 (47 – 86)	78 (56 – 92)	81 (61 – 93)	81 (61 – 93)
Positive LR	3.06 (1.28 – 7.29)	2.73 (1.30 – 5.72)	2.31 (0.84 – 6.36)	2.31 (1.06 – 5.01)	4.86 (0.80 – 29.42)	4.86 (0.80 – 29.42)
Negative LR	0.31 (0.16 – 0.59)	0.21 (0.09 – 0.51)	0.44 (0.18 – 1.09)	0.13 (0.02 – 0.9)	0.23 (0.09 – 0.59)	0.23 (0.09 – 0.59)

The data are presented as % (95 % CI)

**Fig. 5:**
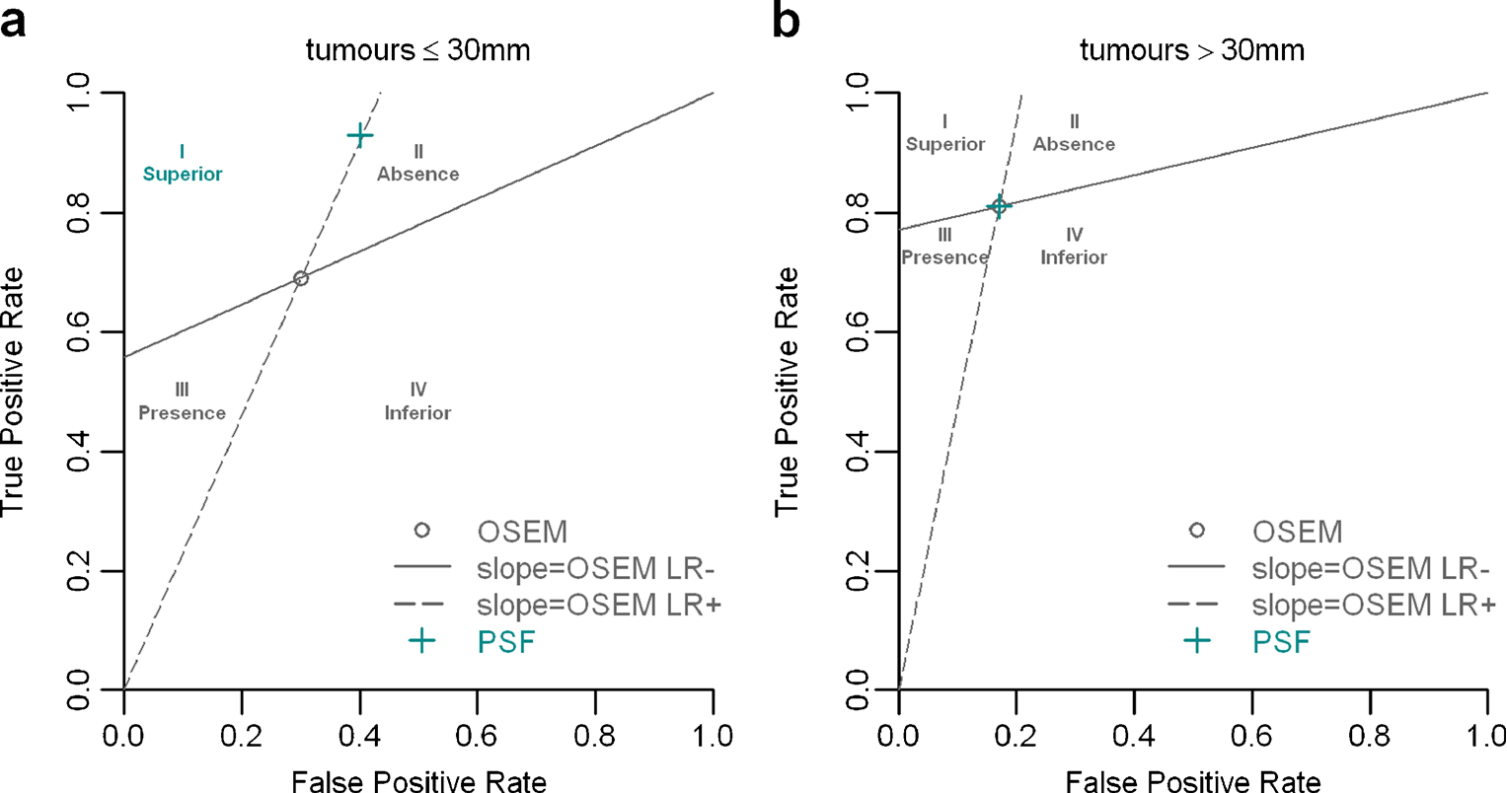
Graphical comparison between the two algorithms. Each subgroup is defined according to the size of the primary tumour (**a** ≤30 mm, **b** >30 mm; 23 and 27 patients, respectively). Compared with OSEM reconstruction, a diagnostic procedure in the first quadrant (*I*) will be interpreted as superior overall, in the second quadrant (*II*) as superior for confirming the absence of metastases, in the third quadrant (*III*) as superior for confirming the presence of metastases, or in the last quadrant (*IV*) as inferior overall

In patients with a primary tumour ≤30 mm, the sensitivity of PSF reconstruction was higher than that of OSEM reconstruction (93 % vs. 69 %) at the expense of a lower specificity (60 % vs. 70 %). However, globally, using the Biggerstaff graphical comparison, PSF reconstruction is superior to OSEM reconstruction (Fig. 5). In patients with a primary tumour >30 mm, both reconstructions performed equally well (Table 2, Fig. 5).

**Fig. 6:**
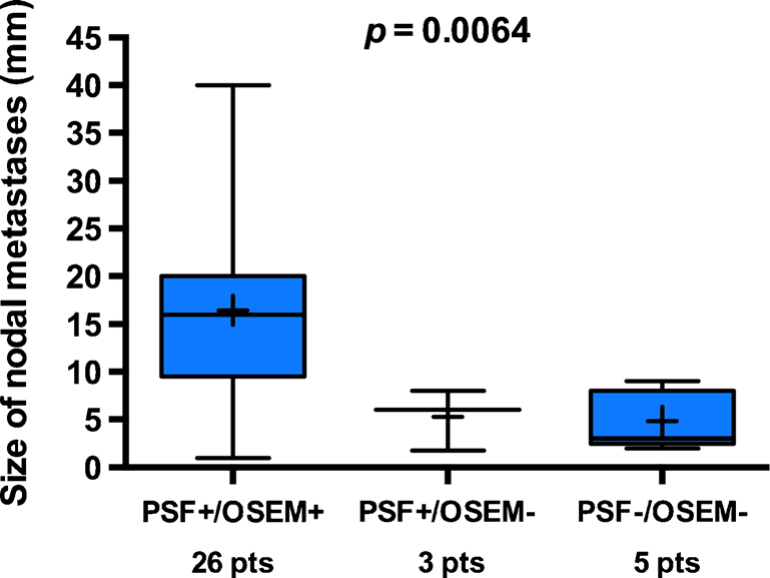
Sizes of the largest nodal metastasis per axilla in relation to nodal involvement status as detected by PET using PSF or OSEM reconstruction: *PSF-/OSEM-* patients with nodal involvement not detected by either reconstruction, *PSF+/OSEM-* patients in whom nodal involvement was detected only by PSF reconstruction, *PSF+/OSEM+* patients in whom nodal involvement was correctly detected by both reconstructions). The extreme values and quartiles (when *n* >3) are shown together with the median values (*longest bars*) and mean values (*shortest bars*). The sizes were compared using the Kruskal-Wallis rank sum test

In metastatic patients with the largest nodal metastasis >7 mm, the sensitivities were 96 % and 92 % for PSF reconstruction and OSEM reconstruction, respectively, and in those with the largest nodal metastasis ≤7 mm, the sensitivities were 60 % and 40 %, respectively. The sizes of the largest nodal metastasis per axilla in relation to nodal involvement status as detected by PET using PSF or OSEM reconstruction are shown in Fig. 6. The median size was 3 mm amongst patients with nodal involvement not detected by either reconstruction, 6 mm amongst patients in whom nodal involvement was detected only by PSF reconstruction and 16 mm amongst patients in whom nodal involvement was correctly detected by both reconstructions. This difference was statistically significant (*p* = 0.0064). In the patients in whom PSF reconstruction detected nodal involvement but OSEM reconstruction did not, the smallest detectable metastasis was 1.8 mm.

## Discussion

Evaluation of new technologies that are being implemented in PET imaging is needed. Some of them such as advanced reconstruction algorithms are likely to not only improve diagnostic performance but also change quantitative and image features, requiring new diagnostic thresholds to be defined. PSF reconstruction is a new reconstruction algorithm available from all major vendors of whole-body PET/CT systems (namely TrueX from Siemens Healthcare [17], SharpIR for GE Healthcare [18] and Astonish TF from Philips Healthcare) which improves spatial resolution and is therefore expected to lead to the detection of smaller metastases than can be achieved by conventional algorithms such as OSEM. So far, PET imaging has failed to solve the clinical issue of proper axillary lymph node staging in breast cancer patients at least in part because of its limited spatial resolution, and could therefore benefit from PSF reconstruction. In this prospective study in a single referral centre, the use of PSF reconstruction led to an increase in SUV_max_ of 66 % on average (Fig. 1) as compared with OSEM reconstruction. PSF reconstruction was able to detect more involved nodes and to improve PET sensitivity in the detection of axillary nodal involvement, especially in patients in whom the largest nodal metastasis was <7 mm.

The implementation of PSF reconstruction improves spatial resolution in a more important manner at the edges of the FOV where the PSF broadens because of the oblique penetration of 511-keV photons into scintillation crystals. Evaluation of the FWHM of our system in the geometry of a human breast and axilla PET examination showed a strong improvement thanks to PSF reconstruction (median radial FWHM: 2.50 mm) as compared to a conventional OSEM algorithm (median radial FWHM: 6.34 mm). In addition, PSF reconstruction minimized PVE and would be expected to improve activity recovery more importantly in lesions smaller than twice the spatial resolution of the PET system. Therefore, the improvement in quantitative values and the ability to detect small lesions would be expected to be higher in the axillae, as compared with more centrally located malignancies and/or larger tumours. Indeed, the 66 % improvement in SUV_max_ in this study can be compared with the 48 % improvement observed in a previous study dealing with thoracic lymph node staging in patients with non-small-cell lung cancer (NSCLC) that used the same PET system and acquisition/reconstruction parameters [8].

In our study, PET with PSF reconstruction detected more involved nodes than PET with OSEM reconstruction . This is of importance, as the number of involved nodes is itself a prognostic factor [19]. In addition, this result strengthens the findings of Vinh-Hung et al. [5], demonstrating that PET may be a powerful tool for distinguishing patients with a low versus those with a high burden of lymph node involvement. PET with PSF reconstruction did not detect involved nodes outside Berg I and II levels that would have been overlooked by PET with OSEM reconstruction . Detecting these nodes, which are usually not addressed during an axillary clearance procedure, is valuable and can change a patient’s management [20].

PSF reconstruction performed better than OSEM reconstruction in detecting nodal metastases <7 mm. We chose the median size of all nodal metastases in our series as a cut-off value, but it is noteworthy that 7 mm was also roughly twice the spatial resolution of our PET system, a size below which PVE is significant. To the best of our knowledge, there is only one study that has compared PET results to the exact size of the intranodal metastases [2]. This is of importance in breast cancer, a malignancy in which a given nodal metastasis is frequently smaller than the involved node by itself. In a recent study evaluating diagnostic full-dose ^18^F-FDG PET/CT for axillary staging of breast cancer patients [2], 10 out of 61 included patients were false-negative. In these patients, apart from an overlooked 24-mm nodal metastasis immediately adjacent to a primary tumour, nodal size ranged from 0.8 to 6 mm (mean 3 mm). In our study, we took into account the size of the largest nodal metastasis per axilla, because this is the lesion most likely to be detected by PET. When taking into account all nodal metastases to compare our results with those of Heusner et al. [2], who used a similar methodology, the size of the metastases in the five patients in whom both PSF and OSEM PET were false-negative ranged from 1 to 9 mm (mean 3.3 mm). In the three patients in whom PSF reconstruction was true-positive while OSEM reconstruction was false-negative, the size of the largest metastases ranged from 1.8 to 8 mm (mean 4.9 mm). The median size of the largest nodal metastasis per axilla was lower in the PSF+/OSEM− group than in the PSF+/OSEM+ group (Fig. 6). Yet in the PSF+/OSEM+ group there were some small metastases (Fig. 6). This illustrates the fact that the ability of PET imaging to detect small cancer deposits depends not only on spatial resolution, but also on other factors such as ^18^F-FDG avidity and contrast between lesion and background.

There is an ongoing debate as to the potential role of ^18^F-FDG PET for initial staging of breast cancer [15, 21–23]. Despite a low sensitivity, ^18^F-FDG PET is generally reported to have a good specificity. Some authors advocate the use of ^18^F-FDG PET to reduce the use of sentinel node biopsy (SNB) [21, 23] (i.e. if findings are positive in the axilla, SNB is no longer required and ALND can be performed immediately), while others suggest that ^18^F-FDG PET should be used to extend the use of SNB [2, 15]. Regarding the latter option, Heusner et al. [2] suggested that in patients with a high risk of axillary lymph node metastases, an unremarkable ^18^F-FDG PET scan could help identify a subgroup of patients who can safely undergo SNB. It is noteworthy that the first strategy is based on a good specificity of PET, generally reported to be higher than 80 %. Similar to the study by Lasnon et al. [8] in patients with NSCLC, we found that PET with PSF reconstruction improves sensitivity at the expense of a slightly lower specificity, probably because PSF reconstruction improves activity recovery in nodes with moderate uptake because of benign disease. In our study, PET with PSF reconstruction had a higher sensitivity in patients with a primary tumour ≤30 mm, whereas both reconstructions performed equally well in patients with a primary tumour >30 mm, a size above which the risk of macrometastases is higher (Table 2). In addition, the LR− was lower with PSF reconstruction than with OSEM reconstruction, and Biggerstaff graphical comparison showed PSF reconstruction to be globally superior to OSEM reconstruction (Fig. 5). Therefore, altogether these data suggest that the use of PET with PSF reconstruction could enable SNB to be performed more safely in patients with a primary tumour ≤30 mm and with unremarkable PET results in the axilla.

Finally, it is noteworthy that this study and others evaluating the impact of PSF reconstruction or a combination of PSF reconstruction and time-of-flight on quantitative values in oncology [7, 8, 24, 25] have been performed at a time when many efforts are being made to harmonize SUV values in multicentre trials [26–28]. The use of different generation PET systems in which FDG PET is used for therapy monitoring in breast cancer patients could lead to inaccurate response evaluation. If, for example, a patient underwent a pretreatment scan on a PET system using a conventional algorithm and a posttreatment scan on a PET system equipped with PSF reconstruction, response would be incorrectly minimized. This may occur in centres running two or more PET systems or updating their equipment during the course of a trial. The use of PSF reconstruction may also be an issue when pooling SUVs coming from PET systems of different generations to determine whether the SUV of metastatic nodes is a prognostic factor [29]. A solution to overcoming these problems is to harmonize SUV using an additional filtering step [30] or by generating two sets of images, one to provide optimal diagnostic quality and the second to meet quantitative harmonizing standards [31].

### Conclusion

In this prospective study in a single referral centre, the use of PSF reconstruction led to an increase in SUV_max_ of 66 % on average compared with OSEM reconstruction, detected more involved nodes and improved PET sensitivity in the detection of axillary nodal involvement, especially in patients in whom the largest nodal metastasis was <7 mm. Although the sensitivity of PET with PSF reconstruction appears to be insufficient for it to replace surgical approaches for axillary staging, our data suggest that the use of PET with PSF reconstruction could allow SNB to be performed more safely in patients with a primary tumour ≤30 mm and with unremarkable PET results in the axilla.

## Electronic supplementary material

Below is the link to the electronic supplementary material.

ESM 1 (DOC 329 kb)
