# Supplementary material for: Staging the axilla in breast cancer patients with 18F-FDG PET: how small are the metastases that we can detect with new generation clinical PET systems?
Source: Eur J Nucl Med Mol Imaging. 2014 Feb 22;41(6):1103–12. doi: 10.1007/s00259-014-2689-7 (PMC4006125; doi:10.1007/s00259-014-2689-7)
Supplement: Supplementary file 1 — (DOC 329 kb) [file 259_2014_2689_MOESM1_ESM.doc]

| **N°** | **Age (yr)** | **Hist*** |  | **Tumour status** | | | | | | | | |  | **Nodal status at pathology** | | | | | | | |  | **Delay between PET and surgery (days)** |  | **PET status** | | | |
| --- | --- | --- | --- | --- | --- | --- | --- | --- | --- | --- | --- | --- | --- | --- | --- | --- | --- | --- | --- | --- | --- | --- | --- | --- | --- | --- | --- | --- |
|  |  |  |  | **cT** | **cN** | **U/B/M*** | **S**  **B**  **R** | **OR** | **PR** | **HER2** | **pT** | **pN** |  | **+/−** | | **Nre*** | **Nin*** | **Nci*** | **Metastasis size (mm)** | | |  |  |  | **OSEM** | | **PSF** | |
|  |  |  |  |  |  |  |  |  |  |  |  |  |  |  |  |  |  |  | median | min | max |  |  |  | +/− | N° of nodes | + /− | N° of nodes |
| 1 | 45 | IDC |  | T3 | N1 | U | III | − | − | + | T2 | N3 |  | + | 20 | | 16 | 0 | 15 | 2 | 30 |  | 1 |  | + | 9 | + | 9 |
| 2 | 73 | IDC |  | T3 | N1 | U | III | + | − | + | T4b | N3 |  | + | 18 | | 10 | 2 | 7.5 | 3 | 20 |  | 28 |  | + | 5 | + | 5 |
| 3 | 50 | IDC |  | T2 | N1 | U | I | + | + | − | T1c | N0 |  | − | 23 | | 0 | 0 | N/A | 0 | 0 |  | 5 |  | − | 0 | − | 0 |
| 4 | 54 | IDC |  | T2 | N1 | U | II | + | + | − | T2 | N0 |  | − | 15 | | 0 | 0 | N/A | 0 | 0 |  | 4 |  | − | 0 | − | 0 |
| 5 | 68 | IDC |  | T2 | N1 | U | II | + | − | + | T2 | N0 |  | − | 10 | | 0 | 0 | N/A | 0 | 0 |  | 5 |  | − | 0 | − | 0 |
| 6 | 70 | IDC |  | T1 | N0 | M | I | + | − | − | T1 | N1 |  | + | 10 | | 1 | 0 | N/A | 3 | 3 |  | 6 |  | − | 0 | − | 0 |
| 7 | 73 | IDC |  | T2 | N0 | U | II | + | + | − | T1b | N1 |  | + | 20 | | 1 | 0 | N/A | 1.8 | 1.8 |  | 43 |  | − | 0 | + | 1 |
| 8 | 54 | IDC |  | T2 | N0 | U | II | + | + | − | T3 | N0 |  | − | 18 | | 0 | 0 | N/A | 0 | 0 |  | 8 |  | + | 3 | + | 3 |
| 9 | 74 | IDC |  | T2 | N0 | U | II | + | + | − | T2 | N0 |  | − | 23 | | 0 | 0 | N/A | 0 | 0 |  | 25 |  | − | 0 | − | 0 |
| 10 | 72 | IDC |  | T1 | N0 | B | I | + | + | − | T1c | N1 |  | + | 10 | | 3 | 2 | 3 | 2 | 8 |  | 13 |  | + | 2 | + | 3 |
| 11 | 58 | IDC |  | T2 | N0 | U | II | + | + | − | T2 | N2 |  | + | 9 | | 4 | 2 | 7 | 0.5 | 20 |  | 5 |  | + | 3 | + | 3 |
| 12 | 51 | IDC |  | T3 | N0 | M | III | − | − | − | T2 | N1 |  | + | 13 | | 3 | 2 | 30 | 4 | 35 |  | 5 |  | + | 3 | + | 4 |
| 13 | 53 | IDC |  | T2 | N0 | U | III | + | + | − | T2 | N1 |  | + | 13 | | 1 | 0 | N/A | 1 | 1 |  | 4 |  | + | 1 | + | 1 |
| 14 | 56 | IDC |  | T2 | N0 | U | III | − | − | − | T2 | N0 |  | − | 22 | | 0 | 0 | N/A | 0 | 0 |  | 5 |  | − | 0 | + | 1 |
| 15 | 62 | IDC |  | T2 | N0 | U | III | − | − | − | T2 | N1 |  | + | 19 | | 3 | 0 | 7 | 7 | 8 |  | 5 |  | + | 3 | + | 3 |
| 16 | 60 | IDC |  | T3 | N0 | U | III | + | + | − | T3 | N1 |  | + | 13 | | 3 | 0 | 10 | 7 | 10 |  | 7 |  | + | 1 | + | 1 |
| 17 | 50 | IDC |  | T2 | N1 | U | II | + | + | − | T2 | N1 |  | + | 9 | | 3 | 1 | 5 | 2 | 20 |  | 5 |  | + | 1 | + | 1 |
| 18 | 45 | IDC |  | T2 | N0 | U | II | + | + | + | T2 | N0 |  | − | 20 | | 0 | 0 | N/A | 0 | 0 |  | 3 |  | − | 0 | − | 0 |
| 19 | 64 | IDC |  | T2 | N0 | U | II | + | + | − | T2 | N1 |  | + | 20 | | 2 | 1 | 5 | 4 | 6 |  | 2 |  | − | 0 | + | 1 |
| 20 | 63 | IDC |  | T2 | N0 | U | II | + | + | − | T2 | N1 |  | + | 6 | | 1 | 0 | N/A | 8 | 8 |  | 15 |  | − | 0 | + | 1 |
| 21 | 74 | IDC |  | T2 | N0 | U | III | + | + | − | T2 | N0 |  | − | 17 | | 0 | 0 | N/A | 0 | 0 |  | 7 |  | − | 0 | − | 0 |
| 22 | 29 | IDC |  | T1 | N1 | U | III | − | − | − | T1c | N1 |  | + | 18 | | 2 | 0 | 25 | 15 | 35 |  | 1 |  | + | 5 | + | 6 |
| 23 | 35 | IDC |  | T3 | N0 | B | III | + | + | + | T2 | N2 |  | + | 20 | | 4 | 0 | 8.5 | 7 | 12 |  | 50 |  | + | 2 | + | 3 |
| 24 | 60 | ICL |  | T2 | N1 | B | III | + | + | − | T3 | N3 |  | + | 15 | | 10 | 4 | 17.5 | 6 | 40 |  | 11 |  | + | 7 | + | 8 |
| 25 | 73 | IDC |  | T2 | N1 | U | II | + | + | − | T2 | N2 |  | + | 10 | | 4 | 3 | 9 | 7 | 15 |  | 23 |  | + | 1 | + | 2 |

*Hist=Histopathology, IDC=Infiltrating ductal carcinoma, ICL=Infiltrating lobular carcinoma, IUC=[Infiltrating undifferentiated carcinoma](http://scholar.google.fr/scholar?q=Infiltrating+undifferentiated+carcinoma&hl=fr&as_sdt=0&as_vis=1&oi=scholart&sa=X&ei=r6uzUIeDIceb0QXQ7oHoAw&ved=0CCoQgQMwAA), Mix.=Mixed (IDC+ICL), U/B/M=Uni-, bi- or Multifocal, SBR=Scarff Bloom Richardson score, OR= Oestrogen receptors, PR=Progesterone receptor, Nre=Number of nodes resected, Nin=Number of nodes involved, Nci=Number of nodes with capsular invasion, N/A=not applicable.

| **N°** | **Age (yr)** | **Hist*** |  | **Tumour status** | | | | | | | | |  | **Nodal status at pathology** | | | | | | | |  | **Delay between TEP and surgery (days)** |  | **PET status** | | | |
| --- | --- | --- | --- | --- | --- | --- | --- | --- | --- | --- | --- | --- | --- | --- | --- | --- | --- | --- | --- | --- | --- | --- | --- | --- | --- | --- | --- | --- |
|  |  |  |  | **cT** | **cN** | **U/B/M*** | **S**  **B**  **R** | **ER** | **PR** | **HER2** | **pT** | **pN** |  | **+/−** | | **Nre*** | **Nin*** | **Nci*** | **Metastasis size (mm)** | | |  |  |  | **OSEM** | | **PSF** | |
|  |  |  |  |  |  |  |  |  |  |  |  |  |  |  |  |  |  |  | median | min | max |  |  |  | +/− | N° of nodes | + /− | N° of nodes |
| 26 | 43 | Mix. |  | T2 | N1 | M | II | + | + | − | T3 | N1 |  | + | 20 | | 1 | 0 | N/A | 6 | 6 |  | 12 |  | + | 2 | + | 2 |
| 27 | 53 | IDC |  | T1 | N1 | B | III | + | + | − | T1c | N0 |  | − | 10 | | 0 | 0 | N/A | 0 | 0 |  | 5 |  | − | 0 | − | 0 |
| 28 | 49 | IDC |  | T1 | N1 | U | II | + | − | − | T1c | N0 |  | − | 11 | | 0 | 0 | N/A | 0 | 0 |  | 14 |  | − | 0 | − | 0 |
| 29 | 45 | Mix. |  | T4b | N0 | U | II | + | + | − | T3 | N2 |  | + | 9 | | 7 | 0 | 10 | 1 | 17 |  | 51 |  | + | 3 | + | 3 |
| 30 | 61 | IDC |  | T1 | N1 | U | III | + | + | − | T1c | N1 |  | + | 10 | | 1 | 0 | N/A | 18 | 18 |  | 5 |  | + | 1 | + | 1 |
| 31 | 56 | IDC |  | T3 | N0 | B | III | + | − | − | T3 | N0 |  | − | 12 | | 0 | 0 | N/A | 0 | 0 |  | 7 |  | + | 2 | + | 2 |
| 32 | 50 | ICL |  | T2 | N0 | M | II | + | + | − | T2 | N1 |  | + | 15 | | 2 | 0 | 3 | 3 | 3 |  | 20 |  | + | 2 | + | 2 |
| 33 | 34 | IDC |  | T3 | N0 | U | II | + | + | − | T3 | N1 |  | + | 7 | | 3 | 1 | 7 | 0.5 | 10 |  | 7 |  | + | 1 | + | 1 |
| 34 | 32 | IDC |  | T3 | N1 | B | III | + | − | + | T1c | N0 |  | − | 23 | | 0 | 0 | N/A | 0 | 0 |  | 12 |  | + | 1 | + | 1 |
| 35 | 63 | Mix |  | T2 | N0 | U | II | + | + | − | T2 | N1 |  | + | 25 | | 1 | 0 | N/A | 2 | 2 |  | 29 |  | + | 1 | + | 2 |
| 36 | 52 | IDC |  | T1 | N1 | B | II | + | + | + | T2 | N1 |  | + | 22 | | 1 | 0 | N/A | 2 | 2 |  | 7 |  | − | 0 | − | 0 |
| 37 | 80 | IDC |  | T2 | N1 | U | II | + | + | − | T3 | N2 |  | + | 20 | | 5 | 3 | 5 | 2 | 12 |  | 20 |  | + | 1 | + | 1 |
| 38 | 61 | IUC |  | T4b | N0 | U | III | − | − | − | T2 | N0 |  | − | 8 | | 0 | 0 | N/A | 0 | 0 |  | 3 |  | − | 0 | − | 0 |
| 39 | 54 | IDC |  | T1 | N0 | B | I | + | + | − | T1c | N0 |  | − | 18 | | 0 | 0 | N/A | 0 | 0 |  | 11 |  | + | 2 | + | 2 |
| 40 | 66 | IDC |  | T2 | N1 | U | II | + | − | − | T2 | N3 |  | + | 16 | | 14 | 3 | 8 | 1 | 17 |  | 3 |  | + | 8 | + | 8 |
| 41 | 32 | IDC |  | T2 | N1 | U | III | + | + | − | T2 | N3 |  | + | 16 | | 13 | 0 | 5 | 1 | 27 |  | 3 |  | + | 3 | + | 4 |
| 42 | 48 | Mix. |  | T1 | N0 | M | II | + | + | − | T2 | N1 |  | + | 10 | | 1 | 1 | N/A | 7 | 7 |  | 8 |  | − | 0 | − | 0 |
| 43 | 51 | IDC |  | T4b | N1 | U | II | + | + | − | T2 | N3 |  | + | 20 | | 15 | 8 | 3 | 1 | 10 |  | 5 |  | + | 3 | + | 5 |
| 44 | 48 | IDC |  | Tx | N0 | U | I | + | + | − | T1c | N0 |  | − | 14 | | 0 | 0 | N/A | 0 | 0 |  | 9 |  | − | 0 | − | 0 |
| 45 | 70 | IDC |  | T3 | N0 | U | III | − | − | − | T2 | N0 |  | − | 12 | | 0 | 0 | N/A | 0 | 0 |  | 5 |  | − | 0 | − | 0 |
| 46 | 68 | IDC |  | T1 | N1 | M | II | + | + | − | T2 | N1 |  | + | 14 | | 1 | 0 | N/A | 20 | 20 |  | 3 |  | + | 1 | + | 1 |
| 47 | 61 | IDC |  | T2 | N1 | U | III | + | + | − | T2 | N1 |  | + | 12 | | 1 | 0 | N/A | 3 | 3 |  | 15 |  | − | 0 | − | 0 |
| 48 | 48 | IDC |  | T2 | N0 | M | I | + | − | − | T1c | N2 |  | + | 24 | | 6 | 2 | 2 | 1 | 9 |  | 5 |  | − | 0 | − | 0 |
| 49 | 63 | IDC |  | T2 | N1 | M | III | + | − | − | T4b | N2 |  | + | 19 | | 7 | 0 | 4 | 1 | 20 |  | 56 |  | + | 1 | + | 1 |
| 50 | 63 | IDC |  | T2 | N0 | U | III | + | − | − | T2 | N1 |  | + | 24 | | 1 | 0 | N/A | 12 | 12 |  | 14 |  | + | 1 | + | 1 |

*Hist= Histopathology, IDC=Infiltrating ductal carcinoma, ICL= Infiltrating lobular carcinoma, IUC= [Infiltrating undifferentiated carcinoma](http://scholar.google.fr/scholar?q=Infiltrating+undifferentiated+carcinoma&hl=fr&as_sdt=0&as_vis=1&oi=scholart&sa=X&ei=r6uzUIeDIceb0QXQ7oHoAw&ved=0CCoQgQMwAA), Mix=Mixed (IDC+ICL), U/B/M= Uni-, bi- or Multifocal, SBR=Scarff Bloom Richardson score, OR=Oestrogen receptors, PR=Progesterone receptor, Nre=Number of nodes resected, Nin=Number of nodes involved, Nci= Number of nodes with capsular invasion, N/A=not applicable.
